# The differential impact of EU attitudes on voting behaviour in the European parliamentary elections 2019

**DOI:** 10.1080/14782804.2024.2356643

**Published:** 2024-05-23

**Authors:** Andreas C. Goldberg, Erika J. van Elsas, Claes H. De Vreese

**Affiliations:** a Department of Sociology and Political Science, Norwegian University of Science and Technology, Trondheim, Norway; b Department of Political Science, Radboud University, Nijmegen, Netherlands; c Amsterdam School of Communication Research, University of Amsterdam, Amsterdam, Netherlands

**Keywords:** European parliament, elections, voting behaviour, EU attitudes

## Abstract

EU attitudes are multidimensional and likely to matter differentially for voting across different parties. The 2019 European Parliament (EP) elections offer a unique setting for testing the differential effects of multidimensional EU attitudes, as the election results entailed increased political fragmentation – with notable pro- and anti-EU party groups in the EP gaining strength. This article examines the importance of EU attitudes on electoral choice and zooms in on the influence of specific EU attitudes on party voting in EP elections (‘EU issue voting’). We use original survey data collected around the 2019 EP elections in ten EU member states, which include a fine-grained measurement of EU attitudes. We find evidence for EU issue voting in all countries, albeit not equally structured across countries. EU issue voting matters across all party groups with affective and performance evaluations having the strongest effects.

## 1. Introduction

Citizens across the European Union (EU) have a diverse range of attitudes towards the EU and European integration. Instead of simply being ‘for’ or ‘against’ Europe, citizens can oppose or criticize the EU on some aspects while embracing it on others. This view of EU public opinion as a heterogeneous, nuanced, and multidimensional whole is increasingly adopted by EU scholars (e.g. Boomgaarden et al. 2011; Hobolt and Brouard 2011; Lubbers 2008). Less is known about the impact of such multidimensional EU attitudes on vote choice in European Parliament (EP) elections. The 2019 EP elections led to further fragmentation of the European party system with the two commonly largest political groups European People’s Party (EPP) and Progressive Alliance of Socialists and Democrats (S&D) having lost their majority in the EP. Other political groups representing different ideological positions, such as the liberal Renew Europe (RE; former ALDE), the Greens/European Free Alliance (Greens-EFA), but also the new Eurosceptic Identity and Democracy (ID) gained in strength. In the words of former European Council (EC) president Donald Tusk, the new EP is ‘a slightly more complex parliament’, which, however, ‘also makes the European Parliament more representative’ and reflects ‘a much greater diversity of views and national sensitivities’.^1^ Given the different EU positions of the EP election ‘winners’, and the increasingly diverse considerations underlying citizens’ voting behaviour (Van der Brug, Gattermann, and de Vreese 2022), this raises the question how EU-related considerations impacted voting decisions.

While there is agreement in the literature that (general) EU attitudes matter for electoral decision-making, including voting for the EP (e.g. Hobolt 2015; Hobolt and De Vries 2016), we know little about *which kind of* EU attitudes matter (most), let alone for which parties. The main goal of this study is thus to investigate the role of multidimensional EU attitudes for EP voting and to zoom in on links between specific EU attitudes and specific vote choice. Our detailed investigation ties into a longer literature on the relevance of EU attitudes for party voting (so-called ‘EU issue voting’), which suggests that EP elections are no longer merely ‘second order national elections’ (e.g. Hobolt and De Vries 2016; Van Elsas, Goldberg, and de Vreese 2019; Van Spanje and De Vreese 2011). In order to run one joint cross-country analysis, instead of separate national analyses, and in the context of increasing similarity of EP political groups with national political parties (McElroy and Benoit 2010), we analyse EU issue voting for parties belonging to different EP political groups.

We rely on original survey data collected around the 2019 EP elections in ten EU member states (CZ, DE, DK, ES, FR, GR, HU, NL, PL and SE). These data include a fine-grained measurement of EU attitudes, which enables the detection of their effects on voting for specific political groups. The comparative analysis across ten countries allows us to address the generalizability of effects. We find evidence of EU-issue voting across all countries under study, albeit to a different extent. The results further show that EU issue voting is not reserved for (smaller) party groups with a more clear-cut pro- or anti-EU profile, but exists across all party groups. Affective and performance evaluations of the EU distinguish most strongly between party preferences, mainly opposing mainstream party groups from radical groups from both the right and left.

## 2. Theory

### 2.1. Changing role of EU issue voting

Over time the description of EP elections as merely second order national elections has changed, as there is increasing evidence of ‘EU issue voting’ (e.g. De Vries and Hobolt 2016; Hobolt 2015). For instance, the surge in support for Eurosceptic parties in the 2014 EP elections was widely interpreted as a reflection of increased public Euroscepticism (Hobolt and De Vries 2016). The growing importance of EU attitudes for electoral behaviour has been interpreted as reflecting the increasing relevance of EU level policy-making. In fact, in contrast to the conventional second-order national election model (Reif and Schmitt 1980) and in line with the original meaning of ‘EU issue voting’, several studies have shown the relevance of EU attitudes for voting in national elections (e.g. Costa Lobo 2023; De Vries 2010; Gabel 2000; Hobolt and Rodon 2020). At the EU level, the growing importance of EU attitudes first became visible in voting for referendums on European integration (e.g. Hobolt 2005; Svensson 2002). More recently, studies have consistently confirmed the importance of EU attitudes on vote choice in EP elections (e.g. Hobolt 2015; Hobolt and De Vries 2016; Van Elsas, Goldberg, and de Vreese 2019), and a recent study comparing the 2014 and 2019 EP elections has documented an increasing importance of EU attitudes on the vote (Carrieri 2024). Even if some aggregate patterns continue to reflect the second-order model (electoral loss of government party, lower turnout as compared to national elections) (Ehin and Talving 2021), individual-level evidence thus points to (increasing) EU issue voting in EP elections. As a result, most scholarship now assumes that there is mileage in both arguments, the second-order model and EU issue voting. Related, the 8% points increase in turnout in the 2019 EP elections might prove to be a break in the continually downward trend in EP elections turnout since 1979, and potentially signals citizens’ increased use of EP elections to voice their opinions about the EU. Including this latter point, the special issue compiled by Gattermann et al. (2021) about the 2019 EP elections equally concludes that there are signs of a more first-order nature of EP elections, also considering other aspects. One such example is the study by Sorace (2021) that shows an association between voters’ party support at the EP elections and respective parties’ level of activism in EP policy-making (as a form of retrospective voting).

While there is thus increasing evidence for a more first-order logic of EP elections, including a general relevance of EU attitudes for EP voting behaviour, our study goes beyond the basic expectation that EU support influences the voting decision by unpacking such ‘EU support’ into more specific EU attitude dimensions. Our main argument is that these specific EU attitude dimensions do not only influence voting behaviour in general but are also relevant to distinguish voting for specific parties. Depending on how a party positions itself regarding the EU, it may draw voters based on particular dimensions of EU attitudes.

### 2.2. The relevance of multidimensional EU attitudes

In contrast to approaching EU attitudes as a one-dimensional concept (e.g. Torcal and Rodon 2021), several studies have demonstrated that citizens’ attitudes toward the EU are best understood as multidimensional (Boomgaarden et al. 2011; De Vreese, Goldberg and Brosius 2023; Hobolt and Brouard 2011). There are different modes (e.g. affective vs. rational) and objects (e.g. the political community, the regime, its institutions or actors) of EU support, and citizens can hold distinct attitudes towards the EU on different attitude dimensions. For instance, a citizen may see the economic benefits of EU membership and thus score high on utilitarian EU support, while at the same time not share a sense of EU identity. Boomgaarden et al. (2011) proposed a five-dimensional model of EU attitudes (utilitarianism, identity, performance, negative affect, and support for EU strengthening). Others have distinguished between regime and policy EU support (De Vries 2018) or between utilitarian, identity-based and political Euroscepticism (Lubbers 2008). Each conceptualisation can have merits depending on the objective of the study and the required degree of differentiation.

As one of the most detailed conceptualisations, we rely on the five dimensions proposed by Boomgaarden et al. (2011), which include more affective and stable attitudes (1) negative affect, 2) identity) and more evaluative and volatile attitudes (3) strengthening, 4) utilitarianism, and 5) performance) with regard to the EU. These five dimensions have been validated both over time and cross-nationally by de Vreese et al. (2019). Furthermore, the relevance of multidimensional EU attitudes has been documented both for EU referendum voting (Goldberg and de Vreese 2018) and for EP election voting (Van Elsas, Goldberg, and de Vreese 2019; Van Spanje and De Vreese 2011), albeit without examining the detailed party choice voters actually faced. Van Spanje and de Vreese (2011) conclude that all five dimensions were relevant in the 2009 EP elections, particularly so the strengthening and utilitarianism dimensions. Van Elsas et al. (2019) confirm the strong effect for EU strengthening in the 2014 elections, and somewhat weaker effects of negative affect and performance evaluations. In a first step and by updating and extending the evidence from the previous 2009 and 2014 elections, we are interested in the overall influence of EU attitudes on party voting in the 2019 elections and the relative importance of the five different attitude dimensions:

RQ1: What is the overall influence of (specific) EU attitude dimensions for the party voting decision in the 2019 EP elections?

### 2.3. Effects of EU attitudes on specific party voting

The just mentioned previous studies on the 2009 and 2014 EP elections were the first to unpack the explanatory part of their models in more detail by including multidimensional EU attitudes. However, none of these studies examined party voting in greater detail. Whereas van Spanje and de Vreese (2011) tested the influence of EU attitudes on Eurosceptic voting behaviour only, van Elsas et al. (2019) grouped parties into pro-, anti- and mixed EU parties. While the latter approach comes closer to the vote choice citizens face, it is still a reduction of the actual choice complexity. Moreover, it does not represent the actual party system of the European Parliament. Studies that unpack specific national party voting such as the one by Torcal and Rodon (2021), in contrast, lack the more detailed focus on multidimensional EU attitude dimensions, as they focus on a one-dimensional EU integration scale. This means that former studies did not allow for a fine-grained analysis of the relationship between specific EU attitudes and specific parties. In this study, we provide such a more detailed analysis by examining to what extent different EU attitudes matter for specific party voting.

Assuming that EU attitudes have an impact on EP voting, it is still unlikely that EU issue voting is equally present across parties – that is, parties may differ in the extent to which they profit or lose on the EU issue. According to issue-ownership theory (e.g. Petrocik 1996), parties take clear positions (positive or negative) on certain issues and emphasize them in their programmes and during campaigns, which may result in parties’ ‘owning’ of certain issues. When these owned issues – including the issue of ‘Europe’ – become politicized, especially parties with strong issue profiles should profit electorally (Kriesi and Sciarini 2004). Given the stronger and more clear-cut position on Europe by smaller pro- and anti-EU parties, these parties should have an electoral advantage compared to their rivals when EU attitudes drive individual vote choice. In contrast, most of the mainstream, established parties often take rather ambivalent or moderately positive positions, as they are torn between the increasing pressures from challenger parties, particularly Eurosceptic ones, and their fundamental commitment to the EU (Adam et al. 2017).

Our study builds upon two key findings of extant research. First, in line with the theoretical expectation of conditional mobilization, the study by van Elsas et al. (2019) comparing pro-, mixed and anti-EU parties finds that EU attitudes matter more for voting for parties with a strong EU-profile (i.e. pro-/anti-EU). Yet, this finding may be partly due to the fact that the mixed category contains both left and right mainstream parties, which draw voters with different EU attitudes (also see Carrieri 2024, who finds EU issue voting to have increased most for Europhile parties in the 2019 EP elections). Second, the *kind* of EU attitudes that drive EU issue voting are dependent upon (left-right) ideology, as these motivations fundamentally differ between radical left-wing and right-wing voters. For instance, comparing left-wing and right-wing Eurosceptic parties, Hobolt and de Vries (2016) find that in the 2014 EP elections, voters for left-wing Eurosceptic parties were mainly driven by economic concerns, while voters for right-wing Eurosceptic parties were motivated by concerns with EU immigration and redistribution policy as well as a general dissatisfaction with the EU. Building on this evidence indicating that party ideology is important for which EU attitude dimensions relate to voting for specific types of parties, we further unpack the dependent variable of EU issue voting – by distinguishing voting for different parties across the whole political spectrum.

In order to facilitate this detailed analysis simultaneously across ten countries, we consider the recoded national voting decision representing the seven EP political groups that officially ran in the 2019 EP elections (or formed shortly thereafter) and are comprising the members of parliament sent from the respective national parties. In other words, although citizens vote for national parties in EP elections and our survey data also captures voting for these national parties as they appeared on the respective ballot sheets (see later data description), we group the national party votes according to their EP political group belonging. The political groups reflect the full spectrum of left-right and nationalist-supranationalist party positions. Over the last decades, EP political groups have become more and more cohesive, powerful, draft their own election manifestos and also formulate concrete policy positions, which means they are now commonly seen as the EU-level equivalent of national-level parties, albeit some differences remain (McElroy and Benoit 2010). Further, following the introduction of the *Spitzenkandidaten* procedure, the respective lead candidates’ EP political group affiliation is becoming more visible, e.g. during campaign events such as Europe-wide broadcasted television debates (Goldberg 2023).

In view of the need to merge national party voting into some cross-country grouping – as one otherwise ends up with a (too) large number of country- or national party-specific hypotheses and related analyses – we consider the EP political groups to better represent the relevant party competition for EP elections than the alternative concept of ‘party families’, in line with McElroy and Benoit (2010). They argue that party family is a static characterisation based on traditional issue associations referring to basic political cleavages. In contrast, the concept of EP political groups is dynamic, as new groups form over time and others cease to exist. Similarly, whereas national parties rarely to never change their party family belonging, they switch their political groups’ membership. This group switching results from (changing) parties’ policy positions, as national parties generally aim to be member of the EP political group which offers the highest policy congruence (see also Bressanelli 2012; McElroy and Benoit 2012). Traditional party families are more heterogeneous policy-wise, especially on more recent issues such as the environment or European integration, which may obfuscate the link between EU attitudes and specific party voting. Finally, while the membership in an EP political group is an official and clear party characterisation, the belonging to a party family is subject of discussion because not all parties have clear positions on classic cleavages, especially problematic for newly formed parties or parties from post-communist member states (McElroy and Benoit 2010).

Following from the presented goal to unpack both the effects of multidimensional EU attitudes and specific party voting (represented by EP political groups), we study the effect of five EU attitude dimensions on seven party groups. While not each of the 35 combinations can be feasibly theorised, we focus on the most likely effects per attitude dimension. Table 1 summarizes our theoretical expectations.

**Table 1.** Expectations for EU attitude effects on party choice.

Political groups EUattitude	EPP	S&D	Renew	Greens-EFA	ID	ECR	GUE-NGL
Negative affect	0/–	0/–	0/–	0/–	++	++	+
Identity				++	––	––	
Strengthening		+		++	––	––	
Utilitarianism	+	+	+	+	––	––	–
Performance	++	++	+	–	–	–	––

Expectations range from strongly positive (++) to strongly negative (––). In case of no expectation, the cell is left empty.

First, an important component of citizens’ attitudes towards the EU is emotional responses. *Negative affect* summarizes feelings of anger, fear and disgust towards the EU. Radical right parties are especially known for their ‘politics of resentment’ (Betz 1993), and their unconditional rejection of EU integration makes them likely to attract voters based on negative emotions. Emotional appeals may also be shared by the GUE-NGL (Group of the European United Left-Nordic Green Left) group from the radical left, yet we know that radical left parties do not fundamentally reject the European project as such, but generally voice a more substantive critique of the ‘really existing EU’ (March and Rommerskirchen 2012). We therefore expect votes for the two radical right groups – ID and ECR (European Conservatives and Reformists) – to be driven by negative affect. Inversely, we expect such negative emotions to have either no or a negative impact on voting for each of the mainstream parties and the Greens – as such parties generally do not mobilize resentment.

Second, feelings of *EU identity* are known to drive EU issue voting. This entails feeling European and identifying with Europe’s history, symbols and with other European citizens. Particularly Green parties can be expected to stand out on this dimension: although traditionally among the more Eurosceptic parties, the Greens have become more supportive of the EU over time, and their EU support increasingly correlates with pro-immigration stances (Jolly et al. 2022), which signals their more cosmopolitan position regarding open borders and a ‘uniting of the peoples’ (Edwards 2008). As such intangible, idealistic positions are captured by feelings of EU identity, we expect this attitude dimension to have a positive impact on voting for the Greens-EFA group. The inverse effect is expected among the radical right party groups ID and ECR, in the light of their fundamental rejection of the EU.

Third, *EU strengthening*, or the furthering and deepening of the European project, has traditionally been a project of mainstream parties. Yet, especially among right-wing mainstream parties, there is increasing ambivalence. Christian-Democratic parties are traditionally a driving force of the European project, yet have moved closer to their conservative allies in the EPP (Hanley 2002). The liberal group (Renew) finds common ground in their support for economic integration yet may internally diverge when it comes to the desirability of more political integration (uniting liberal-conservative (e.g. Dutch VVD, Danish Venstre) and social-liberal (e.g. Dutch D66, Danish Radikale Venstre) positions (see Almeida 2012)). The mainstream left is more pro-integrationist. The S&D have departed from their original protectionist stance towards economic integration, as they increasingly appreciate the possibilities of EU-level economic regulation to curb the negative consequences of market liberalization. For the Greens-EFA, their main issue concern – the environment – is most effectively addressed at the supranational level. Hence, we expect support for EU strengthening to particularly induce voting for S&D and the Greens-EFA. At the other end of the spectrum stand the radical right groups ID and ECR, of which the concerns with safeguarding national sovereignty collide with any form of further integration. Among voters of the radical left GUE-NGL we expect more ambivalence about EU strengthening. As argued above, the radical left critique of Europe is directed at the current EU, not at the idea of EU integration as such (Van Elsas, Hakhverdian, and Van der Brug 2016); further integration steps may under some conditions even be seen as a remedy to perceived neoliberal tendencies.

*Utilitarianism* is about the benefits citizens perceive to derive from their country’s EU membership. Such perceptions are based on cost/benefit calculations, related to both material (e.g. economic) and immaterial (e.g. peace and stability) gains and losses. We expect particularly the ID and ECR groups to attract voters with negative perceptions of the EU’s utility. Such parties often propagate ending or renegotiating EU membership, arguing that their country pays too much, has lost too much of its sovereignty or would be better off in a different international construction. Radical left parties of the GUE-NGL group, too, may be critical of EU benefits, yet their criticism is likely less fundamental for the reasons outlined before. On the opposite end stand the mainstream groups EPP, S&D and Renew, as well as the Greens-EFA. Particularly the mainstream groups, which are deeply intertwined with the process of European integration, are invested in keeping the EU together and thus propagate its benefits. Programmatically, utilitarian EU support is also likely to induce voting for the Greens-EFA, as the EU’s role in climate policy might explain why related Green parties are increasingly EU-supportive in recent years (Jolly et al. 2022).

Finally, the *performance* dimension taps evaluations of the EU’s political functioning – in terms of both processes and policy output. Dissatisfaction with the functioning and performance of the EU likely forms a common ground between the radical right (ID and ECR) and left (GUE-NGL). However, while for the right groups criticism of EU performance is but one of the several negative stances towards the EU – and probably less crucial than concerns with sovereignty or identity – for the GUE-NGL group the functioning of the EU is at the core of its critique. They oppose the EU’s ‘neoliberal’ tendencies, its negative impact on national welfare states and the socio-economic grievances this causes, yet at the same time support ‘a different Europe’ that could remedy these grievances with a more social setup. In addition, calls for a more democratic EU are associated with the left, and such criticism can drive voters of both the radical left (GUE-NGL) and green (Greens-EFA) parties. At the other end, given the mainstream parties’ role as central actors in EU politics (and S&D and EPP in particular), these groups are expected to draw voters who evaluate the EU’s performance positively.

## 3. Data and method

### 3.1. Data

We use original survey data collected in the context of the European Parliament elections in May 2019 across ten EU member states (CZ, DE, DK, ES, FR, GR, HU, NL, SE & PL) (Goldberg et al. 2021). The ten countries represent a variety of smaller and bigger EU member states, geographically spread across Europe and comprising founding members as well as countries from various later EU enlargement rounds. All surveys were conducted by the company Kantar using Computer Assisted Web Interviewing. The country samples stem from Kantar themselves or partner panels such as Lightspeed. Sampling quotas were enforced to ensure nationally representative samples according to age, gender, region and education (checked against information from the National Statistics Bureaus or Governmental sources). The data collection, which was part of a larger research project, followed a panel logic with at least two waves collected in each country. While this panel setup was not necessary for this study, we profited from it as all party voting questions (and several of our control variables) were asked in the final post-electoral wave running from May 27-June 10, whereas our main explanatory variables were asked in the pre-election wave running from April 5–24 (for more details see Table A1 in Appendix). This prior measure of EU attitudes reduces concerns of endogeneity, i.e. that people may first decide to vote for a party and subsequently take over the party’s EU position. While the total number of respondents in the pooled post-election wave is *N* = 12388, the focus on voters only reduces the number of respondents to *N* = 8848 with following country-specific numbers: N_CZ_ = 530, N_DE_ = 868, N_DK_ = 1053, N_ES_ = 942, N_FR_ = 1028, N_GR_ = 1050, N_HU_ = 632, N_NL_ = 806, N_PL_ = 1156, N_SE_ = 783.

### 3.2. Operationalization

Our *dependent variable* is party choice. While the actual survey question asked for the original national parties as written on the electoral ballot, for pooling and comparing the voting behaviour across countries, we recoded all national parties into their EP political group belonging. We use all seven official political groups represented in the European Parliament after the 2019 elections (EPP, S&D, Renew Europe, Greens-EFA, ID, GUE-NGL and ECR). In most cases only one or two national parties belong to the same EP political group, that is, they nicely represent ideological differences across Europe and within the separate countries. In addition, we coded all party voting for a non-affiliated party (NI) or smaller parties not represented in the Parliament into ‘other’ (see Table A1 for a detailed overview).^2^

Our main *explanatory variables* are attitudes toward the EU. We rely on the five attitude dimensions developed by Boomgaarden et al. (2011). The five dimensions comprise attitudes in terms of negative affect toward the EU, identity as a European citizen, strengthening of the EU, utilitarianism toward the EU and performance of the EU. Each attitude dimension is comprised by three survey items measured on a 1–7 agreement scale. After adding the values for the three statements per dimension (3–21 scale) and dividing it by three (1–7 scale), we subtract four to arrive at our final scale measure ranging from − 3 to + 3. The exact wordings for all items per dimension can be found in Table A1. For our main analysis, we use the EU attitudes measured in the pre-election wave, that is, measured before party choice. As a robustness check, and as the campaign period might significantly influence EU attitudes, we repeat the analysis with EU attitude measures from the post-election wave.

As *control variables* we include one measure tapping the second-order perspective by controlling for satisfaction with the national government (Van der Eijk and Franklin 1996) and a second measure controlling for anti-immigration attitudes (McLaren 2006) (combined 5 item scale measure). We furthermore control for left-right self-placement of the respondents plus basic sociodemographics such as education (categorical ES-ISCED coding), age (linear) and sex (female dummy). For all exact wordings and categorizations see Table A1 and for summary statistics Table A2 in the Appendix.

### 3.3. Method

The analysis is based on multinomial logistic regression models, which enables a detailed examination of the influence of EU attitudes on party choice. For the analysis of the overall relevance of EU attitudes on voting behaviour, we run both a full model including all five EU attitudes and separate models with one EU attitude at a time, plus controls and country-fixed effects in every model. Due to correlations of the five EU attitude dimensions, we tested for potential problems of multicollinearity. The resulting variance inflation factors (VIF) are all smaller than four, indicating no multicollinearity problem.^3^ For the specific effects on party voting (EP political group voting) and to ease interpretation of the multinomial logistic models, we present an average marginal effects (AME) plot. Having standardised all explanatory variables in the model, the AME plot displays the increase or decrease in predicted probability to vote for a given political group (compared to voting for another one) for an increase in the explanatory variable by one standard deviation.

## 4. Results

### 4.1. The impact of the five EU attitude dimensions

**Figure 1.**
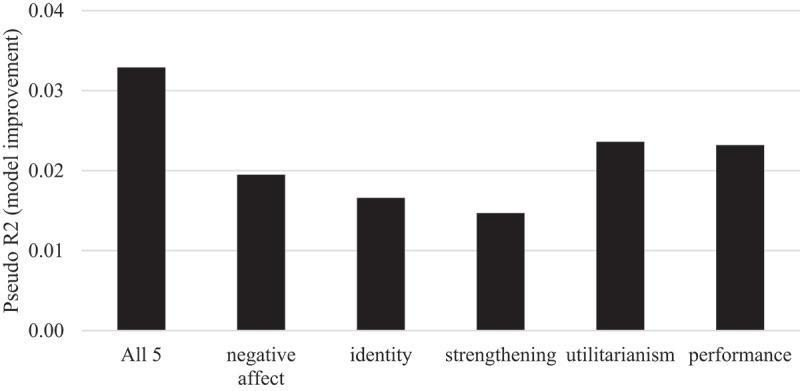
Importance of EU attitudes on party choice.

We begin with the overall importance of EU attitudes on party voting. Figure 1 displays the model improvement by adding all EU attitudes at once or each separately to the baseline model including all control variables (see Table A3 in the Appendix for the full model). In each case, the addition of one or all five attitudes improves the model fit in a significant way (significant likelihood ratio tests). Adding all five EU attitudes at once improves the pseudo R^2^ by around 0.033 (0.292 → 0.325), which represents around ten percent of the overall model improvement.^4^ The cumulative separate dimensions’ model improvement is more than their joint effect, which is due to the relationships between the dimensions (albeit not posing a multicollinearity problem as tested before). Utilitarianism and performance have the strongest influence on party voting, while strengthening is the least important dimension. In sum, we can answer RQ1 by concluding that the specific EU attitudes matter for party voting, albeit with a small to modest substantial effect.

### 4.2. Differences between political groups

**Figure 2.**
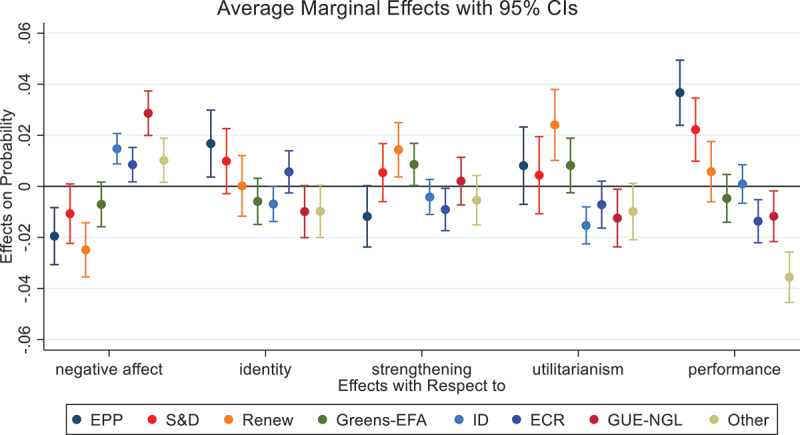
Marginal effects of EU attitudes on party choice.

For the analysis of whether and how EU attitudes distinguish party voting, in terms of distinguishing the different political groups, we consider the results of the pooled multinomial regression results (see Table A3 in the Appendix). To ease interpretation, Figure 2 displays marginal effects for each political group across the five dimensions. Overall, the five attitude dimensions differ in how much they distinguish party voting, with the clearest effects for negative affect and performance and weaker effects for strengthening. Similarly, we also observe differences across EP political groups within several of the dimensions.

Starting with negative affect, we see the expected significant positive effects on voting for extreme parties. Interestingly, these effects are stronger for radical left (GUE-NGL) than for radical right political groups (ID and ECR). We observe no significant effect for the Greens-EFA group. For the three mainstream party groups, in contrast, we observe significant negative effects, albeit significant only on the 0.1 level in the case of S&D. This means that (negative) emotions toward the EU are mobilized in both directions, by mainstream parties as well as by radical parties from both the right and left. The two dimensions of identity and strengthening distinguish party voting less strongly. Feelings of EU identity only have a positive effect on EPP voting, and a negative effect on voting for ID in line with expectations. For strengthening we observe positive significant effects on voting for Renew and the Greens, and a negative effect for ECR, the latter two as expected. However, in terms of substance, the effects of identity and strengthening attitudes in distinguishing party voting are (much) smaller compared to the other dimensions (in line with Figure 1 regarding the overall impact).

**Figure A1.**
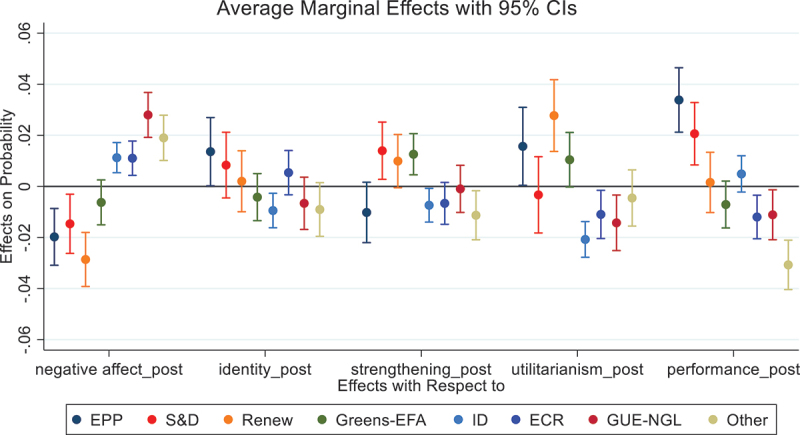
Marginal effects plot with EU attitudes measured in post-election wave.

Regarding utilitarian attitudes, the strong positive effect for Renew stands out in contrast to the two negative effects for ID and GUE-NGL, all in line with our expectations. However, we observe no significant effects of utilitarian attitudes for the remaining party groups. Finally, the pattern of performance shows again stronger and clearer patterns. As expected, we find the clearly strongest positive effects for the EPP and S&D groups, whereas strongly negative effects appear for both radical right and left groups ECR and GUE/NGL. As robustness check, Figure A1 in the Appendix largely confirms the patterns when using the EU attitude values of the post-election wave.

For an easier identification of similarities and differences between the EP political groups, we display all significant effects and their direction in Table 2 (by colouring the respective cells), alongside our theoretical expectations (using the same symbols in the cells as before). While this illustration hides differences in the substantial size of the effects, it allows for an interpretation of the overall patterns and to check whether our expectations are supported. First, it is interesting to note that EU attitudes do not distinguish radical left and right political groups voting more than voting for mainstream groups. This speaks against the conditional mobilization as we do not find that EU issue voting is more relevant for parties with a more clear-cut (anti) EU profile. Quite the contrary, EU attitudes are least relevant for the Greens-EFA and their clear pro-EU profile, with the only significant effect stemming from EU strengthening. It is rather the two more centrist/centre-right party groups EPP and Renew which benefit strongest from pro-EU electoral mobilisation. Importantly, the effects for the mainstream political groups (EPP, S&D and Renew) and the more extreme party groups on the left and right (ID, ECR, GUE-NGL) largely go in opposite directions. Another interesting point is the absence of clear differences between the radical left (GUE-NGL) and the two radical right groups (ECR and ID) in the pattern of effects: for all three radical groups there is a significant effect of the emotional dimension (negative affect), and the radical left is always in line with either of the two radical right groups on the other effects. Finally, while we expected more attitude-party relationships than we found (as indicated by the various uncoloured cells with black plus or minus symbols), none of the found significant effects is opposite to our expectations. Yet the models displayed some few additional effects, e.g. the positive effects for identity on EPP voting and for strengthening on Renew voting.

**Table 2.** Effects of EU attitudes on party choice.

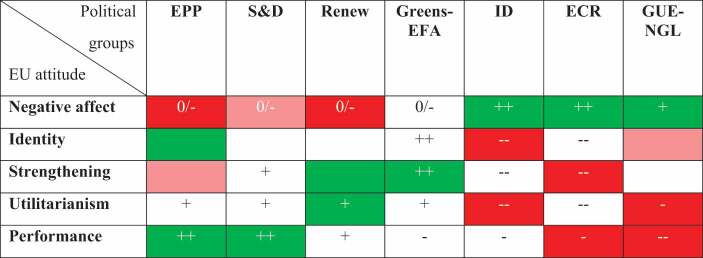

Green cells stand for a positive relationship and red cells for a negative one, with more intense shades representing the 0.05 significance level and lighter shades representing the 0.1 level. In case of no effect, the cell is left uncoloured. The symbols represent our theoretical expectations with all white ones being supported and all black ones not.

**Figure A2.**
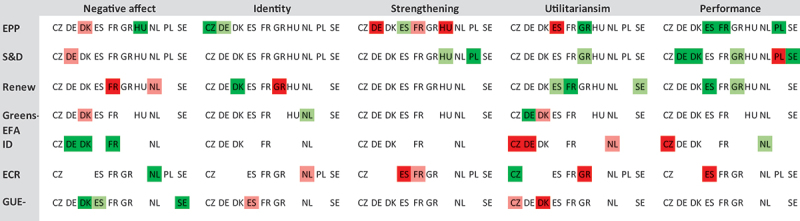
Significance of EU attitude effects on party choice across countries.

As robustness check, we examined whether the party-specific effects found in the pooled model (Figure 2 and Table 2) hold across countries, are driven by single countries only, or whether overall (null) effects are the result of opposing effects for the same political groups in different countries. Figure A2 in the Appendix and related description provides a detailed discussion of these country-specific results. In brief, we find that negative affect and performance have rather consistent patterns across countries. Moreover, in line with the overall weaker effects for the identity and strengthening dimensions, we find mixed and few significant results for these dimensions. Next to these overarching, cross-national patterns, though, we also find some country-specific patterns with partly opposing effects for the same political group across countries. This means that the country-specific constellation of parties and the specificities of competition within countries remain important to understand how EU issue voting manifests itself, next to the presented more general patterns how EU attitudes are linked to party voting across countries.

## 5. Conclusion

This article extends existing literature on ‘EU issue voting’ by unpacking the effects of multi-dimensional EU attitudes for party voting in the 2019 EP elections. We analysed electoral behaviour in a comparative way by examining voting for the seven EP political groups across a set of ten EU member states. In the context of the found EU attitudes’ small to modest substantial effect on party voting, the key conclusion of our analysis is that EU issue voting matters for all EP political groups under study. Overall, we find the comparatively strongest effects for the two dimensions of negative affect and performance, in contrast to earlier studies which found particularly strong effects of the strengthening dimension in the previous 2009 and 2014 EP elections (Van Elsas, Goldberg, and de Vreese 2019; Van Spanje and De Vreese 2011). This indicates that different attitude dimensions matter for different EP elections.

Furthermore, unlike previous research that mainly focused on voting for radical/Eurosceptic parties, our study shows that various attitudes towards the EU impact voting behaviour for *all* EP political groups. In fact, our results speak against the idea of conditional mobilization by parties with a more clear-cut (anti-) EU profile, as both mainstream and more radical party voting are influenced by specific EU attitudes. Interestingly, among the mainstream parties, we found stronger pro-EU attitude mobilisation for the centre/centre-right party groups EPP and Renew, in comparison to weaker than expected effects for the left-leaning S&D and the Greens. In contrast to Carrieri (2024), who finds that proximity on the EU issue (measured one-dimensionally) particularly motivated voting for Europhile parties in the 2019 EP elections, our study’s more fine-grained measurement of EU attitude dimensions reveals that each party group can mobilize specific EU attitudes – and thus profit from EU issue voting. That said, the effects of EU attitudes do differ in strength and direction across EP political groups. For instance, while better EU performance perceptions result in stronger voting for the two mainstream groups EPP and S&D, radical parties on both the right and left profit particularly from stronger (negative) emotional attitudes among voters. These results mean that parties may employ different campaign strategies to benefit from EU issue voting. Focusing on specific attitude dimensions depending on the context of a given election campaign period, e.g. what topics are currently pressing and how these topics may activate certain attitude dimensions, and/or focusing on and activating those dimensions in which a party has comparative advantages over other parties may result in higher voter support.

As a first endeavour into the merits of distinguishing different EU attitude dimensions and different political groups, our study has some limitations that deserve attention. With regard to the independent variable, the inclusion of five EU attitude dimensions allows for a fine-grained analysis of the kind of attitudes that matter to distinguish voting preferences for the political groups – yet is also rather demanding both in terms of modeling and in terms of the assumptions it makes about citizens’ vote decisions. Some level of political sophistication is needed to translate such attitudes into an EP vote decision. Related, while we found support for several of our expected associations between specific EU attitudes and political groups, we also observed some unexpected patterns. Such patterns may relate to internal differences within the EP political groups, which may differ in their homogeneity when it comes to EU positions. These differences may also explain the partly opposing findings when zooming in on the national level. Furthermore, our modelling strategy focused on the influence of EU attitudes to distinguish voting for one party group compared to the other groups, that is, the presented changes in predicted probabilities across parties sum up to zero. This means that our results do not allow to draw conclusions about the impact of single EU attitudes on the *absolute* attractiveness of specific parties. Future research may consider this aspect in more detail, ideally including the whole set of countries participating in the EP elections.

To conclude, voters have attitudes towards various aspects of the EU and European integration, and this variety of attitudes meaningfully impacts their vote decision in EP elections – not only with regard to radical parties but also for the mainstream political groups. Yet, remaining differences across countries remind us that EP elections are to some extent still a collection of parallel elections in the member states, including election campaigns that are mainly run by national parties. Although EP elections result in one common parliament across all EU member states, and although we find systematic voting patterns among the pooled group of countries, this does not necessarily mean that EU issue voting plays out the same way within each country, at least not for the 2019 edition of the EP elections. Yet, in case EP elections become more ‘Europeanized’, in line with developments such as the *Spitzenkandidaten* procedure, European wide parties such as VOLT or an increasing Europeanness of the public debate (Braun 2021), EU issue voting may become (even) more similar across countries in the future. Independent of such future developments, our study is an important step forward in identifying crucial aspects of and differences in ‘EU issue voting’, particularly highlighting the need to consider links between specific EU attitudes and EP political groups. Notwithstanding the partly complex picture our results paint, the main message is that – alongside other aspects – voters take into account different attitudes toward the EU for their decision to vote for specific parties in EP elections.

## Figures and Tables

**Figure A3.**
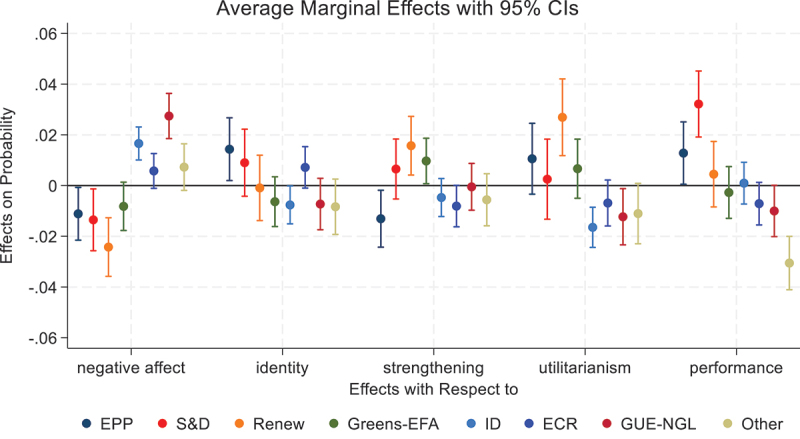
Marginal effects of EU attitudes when excluding voters for parties/alliances attributing seats to more than one party group.

## Data Availability

The data used for the article are freely available at: Goldberg, A.C., van Elsas, E.J., Marquart, F., Brosius, A., de Boer, D.C., & de Vreese, C.H. (2021). Europinions: Public Opinion Survey. *GESIS Data Archive, Cologne. ZA5553 Data file Version 1.0.0*, *https://doi.org/10.4232/1.13795*

## Notes

1.
https://www.consilium.europa.eu/en/press/press-releases/2019/05/28/remarks-by-president-donald-tusk-at-the-press-conference-of-the-informal-summit-of-eu-heads-of-state-or-government/

2. In case of national party alliances or combined lists, the attribution of national party voting to European political groups is partly less straightforward. In case of a clear majority of the received EP seats being attributed to one political group, e.g. Koalicja Europejska (KE) in Poland gaining 17 seats for the EPP and 5 seats for S&D, we recoded all national KE voters into EPP. In case of similar seat shares for two or even more political groups, e.g. CU-SGP in the Netherlands or Ahora Republicas in Spain, we rolled a dice to decide for the linkage to a European political group. Such unclear vote attributions only occurred for five parties/party alliances with mostly only a couple of seats. In the Appendix, we present a robustness check where we exclude all voters for such parties/alliances, without finding significant changes to our main model.

3. The literature offers various thresholds and rules regarding acceptable values of the VIF, e.g. <5 or < 10, without one clear and strict rule (e.g. Field 2014).

4. To put this number into context, running a model including only country-fixed effects results in a pseudo R^2^ of already 0.16, that is, around half of the model improvement is due to country-specific differences. The other half is explained by our individual-level variables. While the contribution of the EU attitudes is smaller than that for the left-right position, EU attitudes account for more model improvement than all the other variables, including satisfaction with the national government and immigration attitudes.

## Appendix

**Table A1.** Overview of variables and their operationalizations.

Variable	Operationalization
*Dependent Variable*	
*Vote choice EP elections*	*Which party did you vote for in the European Parliament elections?* Question was only asked to self-reported voters (separate turnout question) with extensive national party lists plus open ‘other’ option. The recoding of the reported national party options into European political groups results in the following: **EPP (European People’s Party)**: 50Plus, CDA, CDU/CSU, Det Konservative Folkeparti, Fidesz/KDNP, New Democracy (SW), KDU-ČSL, Koalicja Europejska (PO PSL SLD.N Zieloni), Kristdemokraterna, Les Républicains (LR), Moderaterna, Partido Popular, TOP 09/STAN **S&D (Progressive Alliance of Socialists and Democrats)**: Change Movement (KINAL), ČSSD, Demokratikus Koalíció, Feministiskt inititativ, Magyar Szocialista Párt, Parti socialiste, PSOE, PvdA, Socialdemokraterna, Socialdemokratiet, SPD, The River, Wiosna **Renew Europe**: ANO, Centerpartiet, Ciudadanos, Coalición por Europa, D66, FDP, Freie Wähler, La République en marche/Mouvement démocrate/Agir, Liberalerna, Momentum Mozgalom, Radikale Venstre, UDI, Union of Centrists (EC), Venstre, VVD **Greens-EFA (Greens/European Free Alliance)**: B90/Die Grünen, Compromiso por Europa, Die PARTEI, Europe Écologie – Les Verts, GroenLinks, Lehet Más a Politika, Miljöpartiet de Gröna, ÖDP, Párbeszéd Magyarországért, Piraten, Piráti, Socialistisk Folkeparti **ID (Identity and Democracy)**: AfD, Dansk Folkeparti, PVV, Rassemeblement National, SPD (Svoboda a přímá demokracie) **ECR (European Conservatives and Reformists)**: CU-SGP, DLF, FvD, Greek Solution, Independent Greeks (ANEL), ODS, Prawo i Sprawiedliwosc (PiS), Sverigedemokraterna, Vox **GUE/NGL (European United Left – Nordic Green Left)**: Ahora Republicas, Die Linke, Enhedslisten, Folkebevægelsen mod EU, KSČM, La France insoumise, PCF, Piratpartiet, Popular Unity (LAE), PvdD, SP, SYRIZA, Tierschutzpartei, Unidas Podemos, Vänsterpartiet
*Independent Variables*	
EU attitudes (gen. wording)	*Here are a couple of statements about the European Union. To what extent do you agree or disagree with the following statements?* Three statements for each of the following five dimensions presented in a question battery afterwards (all statements in randomized order). The answer scale and procedure to combine the three statements each into a scale was the same for all five EU attitudes. 7-point answer scale with labels for 1 (fully disagree), 4 (neither agree nor disagree) and 7 (fully agree). The values for the three statements have been added, divided by three and finally subtracted by 4 to arrive at the final −3 to + 3 scale.
*EU performance*	The European Union functions well as it is The EU functions according to democratic principles Leaders of member states work efficiently in taking decisions together. *Cronbach’s Alpha: 0.83*
*EU identity*	Being a citizen of the EU means a lot to me. I share a common tradition, culture, and history with other Europeans. The European flag means a lot to me. *Cronbach’s Alpha: 0.80*
*EU utilitarianism*	[NATIONALITY] membership of the EU is a good thing [COUNTRY] has benefited from being a member of the EU The EU fosters peace and stability *Cronbach’s Alpha: 0.88*
*EU negative affect*	I am angry about the EU I feel threatened by the EU I am disgusted with the EU *Cronbach’s Alpha: 0.85*
*EU strengthening*	European integration should go further. The decision-making power of the EU should be extended. The EU should be allowed to collect tax money. *Cronbach’s Alpha: 0.79*
*Satisfaction with government*	*Now thinking about the [NATIONALITY] government, how satisfied are you with the way it is doing its job?* 7-point answer scale ranging from 1 (Extremely dissatisfied) to 7 (Extremely satisfied).
*Anti-immigration*	*Now we would like to hear your opinion on immigrants in [COUNTRY]. Could you indicate for each of the following statements to what extent you agree or disagree with them?* Immigrants abuse the [COUNTRY]’s social welfare system because they take more out than they put in. Immigrants are a threat to the security of [NATIONALITY] people. The religious practices of immigrants enrich the [NATIONALITY] way of life and its traditions. (reverse coding) Immigrants are an important cause of crime in [COUNTRY]. Immigration is good for the [NATIONALITY] labour market. (reverse coding) Combined variable with 7-point answer scale with labels for 1 (fully disagree), 4 (neither agree nor disagree) and 7 (fully agree). *Cronbach’s Alpha: 0.86*
*Left-right*	*Left-right self-placement* 11-point answer scale from 0 (left) to 10 (right).
*Education*	Country-specific educational attainment recoded into ES-ISCED code containing seven categories (recoding followed approach in European Social Survey): 1.ES-ISCED I, less than lower secondary 2.ES-ISCED II, lower secondary 3.ES-ISCED IIIb, lower tier upper secondary 4.ES-ISCED IIIa, upper tier upper secondary 5.ES-ISCED IV, advanced vocational, sub-degree 6.ES-ISCED V1, lower tertiary education, BA level 7.ES-ISCED V2, higher tertiary education, ≥ MA level
*Age*	Birth year recoded into age
*Woman*	Binary variable indicating female respondents (1).

**Table A2.** Summary statistics of survey measures (before standardising them).

Variable	N	Mean/proportion	SD	Min	Max
EU negative affect	8,848	−.820	1.641	−3	3
EU identity	8,848	.112	1.489	−3	3
EU strengthening	8,848	−.411	1.481	−3	3
EU utilitarianism	8,848	.796	1.548	−3	3
EU performance	8,848	−.148	1.386	−3	3
Satisfaction with government	8,848	3.179	1.776	1	7
Anti-immigration	8,848	4.316	1.563	1	7
Left-right	8,848	5.070	2.570	0	10
Sex (woman)	8,848	0.471	.499	0	1
Age	8,848	49.257	15.042	18	99
*Education (ESISCED categories)*					
I (less than lower secondary	8,848	.022	.146		
II (lower secondary	8,848	.094	.291		
IIIb (lower tier upper secondary)	8,848	.289	.453		
IIIa (upper tier upper secondary)	8,848	.056	.229		
IV (advanced vocational, sub-degree)	8,848	.140	.347		
V1 (lower tertiary, BA-level)	8,848	.180	.384		
V2 (higher tertiary, ≥MA level)	8,848	.221	.415

**Table A3.** Multinomial regression results.

	DV: Party choice for						
	EPP	S & D	Renew	Greens-EFA	ID	ECR	GUE-NGL
EU attitude: negative affect	−0.267***	−0.230**	−0.361***	−0.239**	0.217*	−0.031	0.197**
	(0.065)	(0.073)	(0.073)	(0.087)	(0.092)	(0.077)	(0.075)
EU attitude: identity	0.224**	0.167*	0.108	0.004	−0.074	0.225*	−0.034
	(0.076)	(0.083)	(0.084)	(0.095)	(0.105)	(0.092)	(0.088)
EU attitude: strengthening	−0.014	0.148	0.209**	0.241**	−0.016	−0.105	0.132
	(0.071)	(0.076)	(0.077)	(0.088)	(0.105)	(0.091)	(0.081)
EU attitude: utilitarianism	0.178*	0.189*	0.341***	0.265*	−0.232*	0.017	−0.009
	(0.084)	(0.093)	(0.093)	(0.107)	(0.113)	(0.101)	(0.096)
EU attitude: performance	0.603***	0.565***	0.473***	0.347***	0.393***	0.205*	0.242**
	(0.074)	(0.080)	(0.082)	(0.095)	(0.113)	(0.093)	(0.086)
*CONTROLS*							
Government satisfaction (national)	0.054	0.233***	0.484***	0.066	0.005	0.497***	0.343***
	(0.047)	(0.052)	(0.053)	(0.067)	(0.076)	(0.059)	(0.058)
Anti-immigration	0.336***	0.074	0.255***	−0.327***	1.005***	0.768***	−0.041
	(0.057)	(0.060)	(0.063)	(0.073)	(0.090)	(0.075)	(0.063)
Left-right self-placement	0.784***	−0.675***	0.331***	−0.548***	0.561***	1.139***	−1.219***
	(0.055)	(0.060)	(0.060)	(0.072)	(0.076)	(0.070)	(0.067)
Woman	0.084	0.142	0.106	0.376***	−0.142	0.084	0.137
	(0.089)	(0.096)	(0.098)	(0.113)	(0.130)	(0.113)	(0.103)
Age	0.030***	0.032***	0.021***	0.004	0.012**	0.021***	0.016***
	(0.003)	(0.004)	(0.004)	(0.004)	(0.005)	(0.004)	(0.004)
*Education (ref. ES-ISCED I, less than lower secondary)*							
ES-ISCED II, lower secondary	0.357	−0.153	−0.495	−0.292	−0.630	0.223	−0.126
	(0.405)	(0.393)	(0.410)	(0.509)	(0.431)	(0.457)	(0.423)
ES-ISCED IIIb, lower tier upper secondary	0.360	−0.366	−0.202	−0.184	−0.803*	0.354	−0.276
	(0.375)	(0.365)	(0.378)	(0.480)	(0.402)	(0.425)	(0.393)
ES-ISCED IIIa, upper tier upper secondary	−0.096	−0.536	−0.415	−0.039	−0.831	0.108	−0.115
	(0.404)	(0.407)	(0.417)	(0.527)	(0.466)	(0.462)	(0.459)
ES-ISCED IV, advanced vocational,	0.196	−0.367	−0.269	0.008	−0.885*	0.176	−0.236
sub-degree	(0.383)	(0.373)	(0.387)	(0.489)	(0.422)	(0.438)	(0.403)
ES-ISCED V1, lower tertiary education,	0.345	−0.465	−0.232	−0.298	−1.036*	0.041	−0.220
BA level	(0.380)	(0.371)	(0.385)	(0.492)	(0.439)	(0.435)	(0.400)
ES-ISCED V2, higher tertiary education,	0.109	−0.720	−0.196	−0.078	−1.683***	−0.095	−0.395
≥ MA level	(0.376)	(0.368)	(0.381)	(0.483)	(0.436)	(0.427)	(0.398)
*Country dummies (ref. Netherlands)*							
Denmark	−2.016***	−0.939**	−0.724*	−0.628*	0.373	−23.197	−0.623*
	(0.315)	(0.294)	(0.294)	(0.313)	(0.343)	(3646.096)	(0.308)
Germany	−0.908**	−1.535***	−2.049***	−0.001	0.502	−23.190	−0.498
	(0.306)	(0.309)	(0.319)	(0.316)	(0.348)	(5205.771)	(0.318)
Hungary	−1.760***	−1.861***	−2.295***	−3.600***	−22.604	−24.885	−22.989
	(0.289)	(0.291)	(0.293)	(0.379)	(5074.851)	(4226.675)	(4935.238)
Spain	−1.487***	−1.110***	−1.383***	−3.960***	−20.930	−1.782***	−1.166***
	(0.298)	(0.289)	(0.293)	(0.447)	(3937.954)	(0.312)	(0.307)
Czech Republic	−1.379***	−2.708***	−0.752*	−0.119	−0.087	−2.125***	−1.893***
	(0.328)	(0.394)	(0.316)	(0.336)	(0.367)	(0.333)	(0.372)
France	−1.957***	−2.748***	−1.225***	−1.157***	0.574	−3.837***	−1.593***
	(0.296)	(0.305)	(0.286)	(0.306)	(0.332)	(0.362)	(0.308)
Greece	−1.851***	−3.059***	−4.442***	−24.086	−22.914	−3.380***	−1.508***
	(0.275)	(0.288)	(0.334)	(4958.456)	(4409.469)	(0.300)	(0.287)
Poland	−1.057***	−3.513***	−24.471	−24.685	−22.064	−1.172***	−23.774
	(0.276)	(0.295)	(4484.347)	(4799.253)	(5759.414)	(0.281)	(4241.201)
Sweden	0.335	0.198	−0.132	−0.232	−19.776	−0.150	−0.156
	(0.354)	(0.352)	(0.354)	(0.371)	(3835.066)	(0.359)	(0.373)
Constant	0.111	0.854	1.286*	0.825	0.088	0.285	0.403
	(0.497)	(0.493)	(0.504)	(0.600)	(0.579)	(0.558)	(0.531)
Observations	8848						
Pseudo *R*^2^	0.325						

Standard errors in parentheses.

**p* < 0.05, ** *p* < 0.01, *** *p* < 0.001.

### Robustness check: separate multinomial regression model results across the ten countries

Figure A2 summarizes country-specific effects in a simplified graphical form (following the logic of our main results in Table 2). Figure A2 relies on marginal effects stemming from multinomial regression models calculated separately per country (similar to model setup in Table A3). The coloured cells stand for significant marginal effects of a given EU attitude on the voting choice for party belonging to the respective political group. Red stands for negative effects and green for positive ones. The intensely coloured cells represent effects significant at the 0.05 level and the lighter cells at the 0.1 level. In case of empty cells, i.e. no country abbreviation, the respective political group was not represented in this country.

Starting with the effects for negative affect, the country-specific results match the pooled effects well. The positive effects on radical groups can be found in various countries, especially so in Denmark. The significant negative effect for Renew seems to be especially driven by the French voting for Macron’s LaREM. Interestingly and despite the overall negative effect for EPP, only in Denmark we observe a significant effect on the country level (Det Konservative Folkeparti), with an even opposing effect in Hungary (Fidesz/KDNP). In line with the overall weaker effects for the identity and strengthening dimensions, we find few country-specific effects for these dimensions. For the identity dimension, the opposing effects on voting for Renew is noteworthy, with negative effects in Greece (Union of Centrists (EC)) and positive ones in Denmark (Venstre and Radikale Venstre). These opposing effects may explain the absence of an overall effect in the pooled analysis. The general negative effects of strengthening attitudes for voting EPP and ECR are supported by various country-level findings, despite an again slightly opposite effect in Spain for EPP (Partido Popular). Noteworthy is the absence of an effect of strengthening for Renew in any country, despite a general positive effect in the pooled model.

For the utilitarian dimension, we observe several country-level effects, which are mostly in line with the pooled results. While both the positive effect for Renew and the negative effect for ID can be seen in various countries, the opposing significant effects for EPP, Greens-EFA and ECR in two countries each were hidden in the pooled effects. This dimension thus demonstrates how the effects of EU attitudes on (same) party voting may strongly differ across countries. Relying on pooled models only, one would miss these important country differences. In contrast, for performance, the country-specific effects are highly consistent, especially for EPP and S&D voting, albeit the deviating S&D effect in Poland (Wiosna). The absence of a significant overall effect on ID voting may be due to opposing effects in single countries such as the Czech Republic (SPD (Svoboda a přímá demokracie)) and the Netherlands (PVV).

### Robustness check: multinomial regression model that excludes voters for parties/alliances with seat attribution to more than one party group

Figure A3 displays average marginal effects from a multinomial regression model that excludes all voters for parties/alliances that attributed their won seats to more than one party group (or to one party group and a non-affiliated seat). This concerns the Dutch CU-SGP (N = 65 voters), the German Die PARTEI (N = 26), the Spanish Unidos Podemos (N = 133) & Ahora Repúblicas (N = 28) and the Polish Koalicija Europejska (N = 515), in total 767 of the original N of 8848. Given that the clearly largest share of votes, the Polish KE votes, were assigned to the EPP in our main model (as they received 17 out of the 22 EP seats, and the remaining 5 seats were attributed to the S&D), the below plot shows mainly differences for EPP effects. Yet, the overall pattern as discussed in the main text remains unchanged.
